# Radiographic Assessment and Chair Time of Rotary Instruments in the Pulpectomy of Primary Second Molar Teeth: A Randomized Controlled Clinical Trial

**DOI:** 10.5681/joddd.2014.015

**Published:** 2014-06-11

**Authors:** Abbas Makarem, Navid Ravandeh, Masoumeh Ebrahimi

**Affiliations:** ^1^Professor, Department of Pediatric Dentistry, Dental Research Center and Dental School of Mashhad University of Medical Sciences, Mashhad, Iran; ^2^Pedodontist, Private Practice, Tehran, Iran; ^3^Associate Professor, Department of Pediatric Dentistry, Dental Research Center and Dental School of Mashhad University of Medical Sciences, Iran

**Keywords:** Primary second molars, pulpectomy, rotary system

## Abstract

***Background and aims.*** The superiority of rotary systems has been reported in several clinical studies on permanent teeth. This study consisted of radiographic assessment and chair time of rotary instruments in the pulpectomy of primary second molar teeth.

***Materials and methods.*** In this randomized controlled clinical study, 46 children, 3-6 years of age, were selected. The patients were divided randomly into two groups. In the first group (group A) pulpectomy was carried out with hand instruments and in the second group (group B) the Rotary FlexMaster System was used. T-test and chi-squared test were used to analyze data.

***Results.*** The mean instrumentation time in group A was significantly more than that in group B (P<0.001). Also there was a significant difference between both groups in relation to the distance between the apex of mesial root (P<0.001) and distal root (P=0.007) and the canal filling level.

***Conclusion.*** Superior radiographic findings and less chair time of pulpectomy with rotary instruments in second primary molar teeth were achieved.

## Introduction

When extensive caries affects the tooth pulp, pulp therapy becomes necessary. If pulpal inflammation extends to the root canal, then a pulpectomy procedure is indicated. One of the limitations of pulpectomy in primary teeth is the long chair time during which the child’s behavior management can be problematic.^1^

Traditionally, stainless steel files have been used for cleaning and shaping the primary tooth root canal.^2^ Recently, various types of rotary systems have been introduced. Superior characteristics of these alloys are shape memory, high elasticity, higher strength and lower modulus of elasticity compared with stainless steel files.^3,4^

The superiority of rotary systems has been reported in several clinical studies on permanent teeth.^5-9^ Advantages such as maintenance of the canal curvature,^6,8,10-16^, reduced working time,^6,11,12,14,17^ minimal likelihood of instrument fracture ^6,7,11^ and ideal obturation form^6,12^ have been attributed to using rotary instruments.

The FlexMaster (FM) system has been shown to be highly efficacious when used on permanent teeth.^7,8,10,11,14-16,18^ Although successful results have been reported with rotary instruments in experimental studies in primary molars,^2,3,19-21^ limited clinical studies have been reported to date on primary teeth about the FlexMaster (FM) system.^22,23^ The purpose of this study was radiographic assessment and chair time of rotary instruments in the pulpectomy of primary second molar teeth.

## Materials and Methods

This clinical trial enrolled 46 children, 3 to 6 years old, who had extensive caries in their primary second molars and a history of spontaneous pain. Clinically, there were no acute or chronic abscesses. They were referred to the Department of Pediatric Dentistry of Mashhad Dental School from January to June 2007. The Ethics Committee of Mashhad University of Medical Sciences approved this research (#84143); only children with informed consent from their parents were evaluated in this study.

Forty-six children (23 in group A, 23 in group B) were selected for this randomized single- blinded clinical study. Trial design was parallel with 1:1 allocation ratio. Random allocation sequence was done with random table by a statistical consultant.

For the first session, prophylaxis with fluoride therapy was carried out and the children’s level of cooperation was evaluated. According to Wright’s clinical classification, only cooperative children were selected for participation in the study.^1^ Periapical radiographs of the primary second molars were taken by a pediatric dentist using the paralleling technique with the Villa Dental X-Ray System. The patients’ position and horizontal and vertical radiographic angles were standardized to achieve comparable before-and-after images. Only the teeth without internal or external resorption or radiolucency in the furcation or periapical area were selected for the present study.

In the first group (group A), pulpectomy was carried out with hand instruments, and in the second group (group B) the FlexMaster (FM) system was used.

### Conventional Method (group A) 

In group A, after obtaining access, the pulp tissue in the mesial and distal canals was removed using broaches (#20) based on working length, which was measured from the tips of the mesial or distal cusps to 2 mm from the apex. Then the canals were instrumented with Hedeström files #20, #25, and #30.

### Rotary FM System (group B)

In group B, the rotary FM system was used. These instruments have a convex cross-section without radial lands, a negative cutting angle, and three cutting edges.^18^ As in group A, working length was measured from the cusp tips to 2 mm from the apex, using the crown-down preparation technique.

The sequence of FM instrumentation was as follows: (1) 4% taper, #20; (2) 4% taper, #25; and (3) 4% taper, #30. After each step, a file (#15) was used to maintain working length. Based on manufacturers’ recommendation, no file was used for more than 5 seconds. After each reaming, the root canals in both groups were irrigated thoroughly with normal saline solution. The instrumentation time was recorded with a chronometer in both groups until bleeding in the canals ceased. After instrumentation, the canals were dried with paper points (#30 and #35).

Then the canals were filled with a creamy paste of zinc oxide and eugenol (ZOE) using a Lentulo spiral (#25) mounted in a low-speed handpiece. Then the duration of canal filling was registered for both groups. Zinc phosphate cement was placed over the ZOE. Then restorations (stainless steel crown or amalgam fillings) were completed depending on the amount of dental tissue remaining.

Final radiographs were taken by the same operator rendering treatment and marked according to a random selection of patients’ file numbers. Two other pediatric dentists, who were blinded to assignment and intervention, evaluated radiographs after pulpectomy. The radiographs were evaluated using a magnifier and a viewer box. The level of inter-examiner agreement in evaluating the radiographs was good. Consequently, the following parameters were recorded for both groups:

- Mean distance from apex to fill level in mesial roots
- Mean distance from apex to fill level in distal roots
- Obturation form
- Presence of a perforation

Obturation form was divided to acceptable and non-acceptable according to presence or absence of void in the final radiographs.

The mean distance of the apex to the fill level of the mesial and distal roots was evaluated qualitative and classified into superior, fair and poor categories. *Superior* was defined when the distance of the apex to the fill level was 2-2.5 mm. *Fair* indicated that this distance was 2.5-3.5 mm. Poor quality was considered to be a distance greater than 3.5 mm.

The data were analyzed using SPSS version 11.5. Kolmogorov-Smirnov test showed normal distribution of parameters about instrumentation time of canals and distance of apex to fill level of mesial and distal roots. Independent-samples t-test was used for data analyzing of instrumentation time of canals and distance of the apex to the fill level of the mesial and distal roots. Chi-squared test was used for qualitative evaluation of distance of the apex to the fill level in canals and obturation formin both groups. Statistical significance was based on probability values <0.05.

## Results

The mean age was 5.2±0.7 years in the study group. By group, mean ages were 5.12±0.67 and 5.2±0.88 in groups A and B, respectively. Mean instrumentation time was 18.73±3.15 minutes in group A and 10.1±1.71 minutes in group B. Student’s t-test showed significant differences between groups in terms of instrumentation time. However, there were no differences in terms of canal obturation time with ZOE between the two groups. As a result, in the rotary group (A), chair time (instrumentation time + obturation time) was significantly less than that in hand instrumentation group (B) (P<0.001) (P=0.245) (Table 1).

**Table 1:** Mean and standard deviation of studied variables for the conventional and rotary methods

Variables	Conventional method		Rotary method		P
	Mean	SD	Mean	SD	
Instrumentation time	18.7	3.15	10.1	1.71	<0.001
Canal filling time	2.85	0.53	2.69	0.4	0.245
Distance between apex mesial root to canal filling level	3.13	1.1	1.96	0.77	<0.001
Distance between apex distal root to canal filling level	3.1	1.2	2.17	0.98	0.007

The mean distance from the apex to the filling level of the mesial root canals was significantly greater in group A (3.13 mm) than that in group B (1.96 mm) (Student’s t-test, P<0.001). As shown in Table 1, the distance of the apex to the filling level in the distal root also differed significantly between group A (3.1 mm) and group B (2.2 mm; P=0.007).

Rotary method yielded the following categories for mesial canal: superior (82.6%), fair (13%) poor (4.4%). However in conventional method, 30.4% were in the superior category, 39.2% in the fair category and 30.4% in the poor category. The results showed a significant difference between both groups (P=0.0015). Regarding the mean distance between the canal filling level and the apex of mesial roots in rotary group, there was a significant difference between the three categories (superior, fair, poor) (P=0.0015) (Table 2).

**Table 2:** The frequency distribution (%) of distance between the apex of mesial roots and canal filling level in the study groups

Group	Conventional method	Rotary method	P- Value
	N (%)	N (%)	
Superior	7 (30.4%)	19 (82.6%)	
Fair	9 (39.2%)	3 (13%)	
Poor	7 (30.4%)	1 (4.4%)	0.0015
Total	23 (100%)	23 (100%)	

Analyzing the adequacy of filling in the distal canals showed no significant differences between conventional and rotary methods (P=0.986) (Table 3).

**Table 3:** The frequency distribution (%) of distance between the apex of distal roots and canal filling level in the study groups

Group	Conventional method	Rotary method	P
Superior	9 (39.1%)	16 (69.6%)	—
Fair	6 (26.1%)	4 (17.4%)	—
Poor	8 (34.8%)	3 (13%)	0.986
Total	23 (100%)	23 (100%)	—

There were significantly more superior mesial canals in the rotary group (B) than the hand instrumentation group (A) (P=0.0015). However, in the distal canals, the difference was not significant (P=0.986) (Tables 2and 3).

Chi-squared analysis showed that obturation in the rotary group had less voids than the other one; the inter-group difference was significant (P=0.0017) (Table 4). Also, no perforations were seen in either group.

**Table 4:** The frequency distribution (%) of obturation form according to filing method in the study groups

Obturation form	Conventional method	Rotary method	P value
Acceptable	11 (47.8%)	17 (73.9%)	—
Non acceptable	12 (52.2%)	6 (26.1%)	0.0017
Total	23 (100%)	23 (100%)	—

## Discussion

In the present investigation, the FM system with a 0.04 taper was compared with stainless steel files for efficacy in terms of instrumentation time, canal filling time, obturation form, canal filling quality and root perforation. This study showed that mean canal instrumentation time was significantly lower in rotary method than conventional method, agreeing with Barr'^2^ and Silva's^21^ findings, indicating that primary molar pulpectomy using rotary method reduces chair time. This advantage is of prime importance for children who are less cooperative, patients whose dental treatment is carried out under general anesthesia, and children who cannot tolerate multiple appointments. It is also essential to decrease the working time for children having several teeth in need of pulpectomy. Several prior studies on permanent teeth have shown decreased canal instrumentation time with rotary system;^7,10-14^however, up to now, there has been no clinical study on primary teeth in Mashhad Dental School.

There were no differences between the two groups in canal filling time (with ZOE using the Lentulo), indicating that instrumentation with rotary system had no effect on filling time of the canals.

In rating the completeness of canal filling, we divided the results into three categories based on the distance from the apex to the filling level. The higher proportion of superior canal filling in the rotary method relative to conventional method (0.0015) might be attributed to better preparation of canals with the rotary method. Better preparation of canals with the rotary method results in better flow of filling materials into the canals. As mesial canals are usually narrow, their preparation is more difficult when hand instruments are used.

We noted that when the mesial canals were instrumented with the rotary method, the probability of the results falling into the superior group was high, which might be due to better canal instrumentation in this method. In the conventional method group, this variable did not differ significantly between the three categories (P=0.381). Hence, mesial canal filling with the conventional method had similar probabilities of resulting in any of the three categories.

Considering the large distal canals in 3-6-year-olds, it appears that canal preparation with rotary method had no great effect on filling the distal canals in comparison with the conventional method (P=0.986). In relation to the integrity of canal obturation with the rotary method, however, there were significant differences between the three categories (P=0.004). In other words, with the rotary method it was highly probable that the distal canal filling would be superior; nearly two-thirds of the subjects were in this category, which is favorable. With the conventional method, there were no significant differences between the three categories (P=0.068); therefore, the probability that the distal canal filling would be represented in every category is nearly identical.

Regarding obturation form, 73.9% in rotary group and 47.8% in conventional group were acceptable, with a significant difference between groups (P=0.0017). In the present study, the probability that the filling followed the canal form was 47.8% (or 52.2% that it did not) with conventional method, whose difference was not significant (P=0.67). In other words, with the conventional method, it is possible that the filling would follow the canal form. However, in the rotary group, 73.9% of the fillings followed the canal form, whose difference was significant (P=0.002). As a result, consistent with other studies,^10,14^the canal forms were more closely followed with the rotary method in the present investigation. In several studies, maintaining the canal form was attributed to the high flexibility of FM instruments.^10,14^

In this study, no canal perforation was reported in either group. In a study by Cheung, more perforation incidents were reported in association with using conventional method compared with the rotary method.^8^ It should be considered that type of randomization was single-blinded, as one operator performed pulpectomy in both groups; this was a restriction in the present study.

We hope that continuing improvements in rotary systems will result in more efficient and effective treatment of primary teeth in the future.

## Conclusion

Pulpectomy procedure with rotary method reduces chair time in primary molar teeth. In addition, obturation quality with rotary system is more acceptable than the conventional method.
